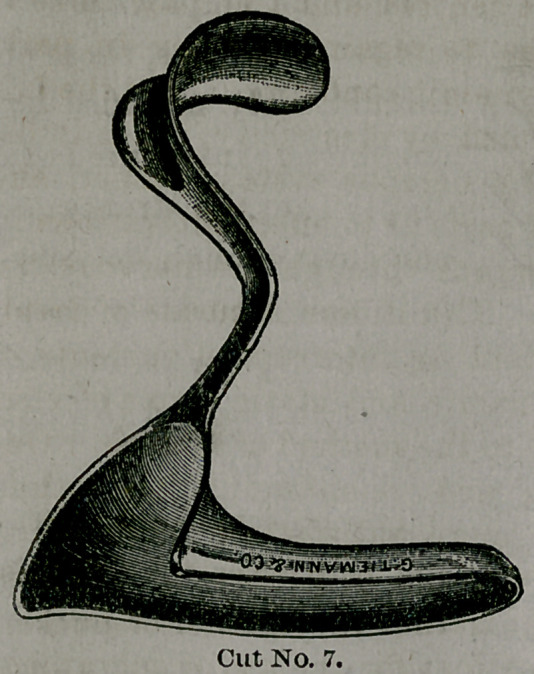# Taliaferro’s Hard Rubber Intra-Uterine Stem Pessary, Retro-Displacement Pessary, Universal Pessary and Tracheloraphy

**Published:** 1883-09

**Authors:** Geo. H. Noble

**Affiliations:** Atlanta, Ga.


### TALIAFERRO'S HARD RUBBER INTRA-UTERINE STEM PESSARY, RETRO-DISPLACEMENT PESSARY, UNIVERSAL PESSARY AND TRACHELORAPHY.

BY GEO. H. NOBLE, M.D., ATLANTA, GA

In presenting to you these instruments for Prof. V. H. Taliaferro, I shall call attention first to his Hard Rubber Stem Pessary, the most marked feature of which is its form.

Cut No. 1.

Being a *thin flat* strip of hard rubber it does not disturb the normal relations of the walls of the flattened cervical canal. It does not revolve upon itself, as the round stems do. Neither does it have the up and down motion to the extent of other instruments, as its flattened shape and elasticity enables it to cling more closely to the parts.

Its lightness, elasticity, and perfect adaption makes it as little objectionable as possible as a foreign substance to the cavity of the uterus.

It therefore is considered the most rationally constructed and safest instrument of its kind.

TALIAFERRO'S RETRO-DISPLACEEMENT PESSARY.

This is but a modification of the Smith-Hodge pessary, being altered so as to conform more closely to the anatomical structures, and to utilile the constriction in the vagina, pointed out by Dr. Sims.

Cut No. 2.

The distal extremity is much more expanded than the other, with a slight depression or bending back at the extreme end, (not shown in cut), which is intended to serve as a double curve, the one applying itself to the roof of the posterior cul-de-sac and the other adapting itself to the transverse curve of the uterus. The action of this being to brace the uterus and to prevent its inclination to one or the other side as so often occurs with the ordinarily constructed pessaries.

When this instrument is in position the supra vaginal constriction of Sims closes around it below the expanded oxtremity, holding it in situ, and at the same time giving it an upward tendency.

It is not necessary with the pessary, then, for the proximal extremity to reach to the pubic bone as a point of support, but should fall short short of it an inch or more. The result is that the uterus is permitted far greater mobility than with the ordinary instruments, with the minimum risk of concussion.

TALIAFERRO'S UNIVERSAL PESSARY.

This instrument is peculiar in shape, resembling on the one hand a cradle pessary, and upon the other the foregoing implement with an exaggerated curve.

Cut No. 3.

The side view is very much like the letter U, with the arms a little separated at the top.

Upon the distal end we have the same double curve or depression as mentioned in the preceding pessary, which, of course, serves the same purpose. To explain the action of this appliance it will be necessary to give its points of bearings, or its relations to the vagina, etc. That portion represented by the bottom of the U bears upon the perineal body and posterior wall, the proximal extremity curves a little upwards and rests lightly against the anterior wall about midway the vagina, while the distal end is applied to the posterior cul-de-sac.

Cut No. 4.

The sides of the pessary consequently rest upon the floor of the pelvis on either side of the vaginal orifice, and it is thereon that it plays in its rocking motion. For if we conceive of a weight being applied to the ulterior arm, it will sink down, while the other arm or extremity of the pessary will rise up, acting precisely as the rocker of a chair. Then when it is relieved of the superincumbent weight it will rock back to its normal position.

So, this action, in addition to the upward and downward motion in the vagina that this and other instruments have, give the uterus a very much greater freedom of mobility. For after this pessary has come down to its lowest limits it tilts or rocks back, permitting the womb to drop lower and safer downwards and backwards. So, then, the Doctor claims less resistance to the physiological movements of the uterus than is offered by other pessaries. And a most admirable feature in it is, that with it we get no shock or concussion when the woman coughs or suddenly strains. Though this is designed as a universal pessary, applicable alike to posterior and anterior displacements, it is not intended to supplant all others, but is especially recommended in difficult cases when others fail. It is especially adapted to cases where there is a loose, lax, and sub-involuted condition of the vagina; where there is a low or partial loss of the perineal body, and where there is a want of tonicity of the parts from frequent child-birth. While this in the main is correct, it is often used in the virgin cases of prolapsus, anterior and posterior displacements, with the best results.

With it, as with all other pessaries, its fit should be perfect. By perfect we mean, that it should correct the mal-position, and at the same time permit the most perfect freedom of all the physiological movements of the uterus. It, perhaps more than any other, should fit so as to have free and easy motion to attain its best results; and should hug the cervix posteriorly and anteriorly, its proximal arm reaching but little in front of the cervix. The patient with it, as with any other, should be taught the removal and re-introduction, which she should daily practice. Unless this be done no woman is free from danger with this or any other pessary.

The objection to the pessaries hitherto used is their obstruction to the free physiological mobility of the uterus, and it is with this end that this pessary is presented to the profession.

The instrument in cut No. 4 is represented as too long and as extending too far upon the anterior vaginal wall.

### TALIAFERRO'S TRACHELORAPHY.

### A MODIFICATION OF EMMET'S OPERATION FOR LACERATION OF THE CERVIX-UTERI.

Dr. Taliaferro has repeatedly found an objection to Emmett's operation in the strain or tension put upon the sutures. He finds that it frequently causes them to cut into the tissues, allowing the lips to separate, thereby necessitating a cicatrix to a greater or less extent—a thing, which when practicable, should always be avoided—and that the union does not take place in the wound as deeply as is desired to form a firm and serviceable cervix. So he has conceived an idea, calculated to overcome this twofold obstacle, and to close the wound perfectly along the margin of the cervical canal. This he does by passing through the cervix antero-posteriorly, from two to three (sometimes only two) wires on either side of the canal so as to emerge from, and re-enter the denuded surfaces, close to the border of the mucous membrane. (See *a a* cut Nos. 5 and 6.)

These are secured by slipping on to them a small leaden plate about 3-16 inch, or ¼ inch in diameter, with an aperture in the centre large enough to admit the wire: and then a perforated shot is made fast by compression. It will be found convenient to do this before introducing the wire.

The plate is intended to give greater bearing surface, the object of which is obvious in itself.

By this we see the greater part of the tension is put upon these wires, which closes the wound effectually along the cervical canal (as is seen in cut No. 6, at *e*, on next page).

Cut No. 5.

Then he employs a second set of sutures, those of Emmet, only modified by not being introduced so deeply into the structures, but just enough to carefully and accurately coaptate the lips or outer borders of the wound (*b b* cut Nos. 5 and 6).

The objection to inserting the wires too deep is that they gather or pucker the surface of the denudation, as does the drawing-string of a bag (though not to such an extent). This is illustrated in cut No. 6, by the puckering of the tissues within the wire (see *c d*), as is ordinarily inserted.

Cut No. 6.

Cut No. 6 represents a transverse section of the cervix showing the position of the sutures. Those on the right are in the Taliaferro modification. The margins of the paired surfaces along the canal illustrate them as they are closed, which not only gives the good results of perfect coaptation, but prevent such secretions as may take place in the cavities of the uterus and cervix from forcing themselves between the lips of the wound. This has been demonstrated in the case of cervical endo-metritis. Those on the left show the deep suture and corrugation of tissues, as is fcund in the ordinary operation. By applying the denuded surfaces one to the other more extensively and eorrectly, we get better and firmer union; but this can only be done with the deep suture of Prof. Taliaferro.

Cut No. 7.

Cut No. 7 is a recently improved perineal elevator, used by Dr. Taliaferro in making this operation, and in tamponing the vagina. This instrument has been modified since first presented to the profession some twelve months since, simply by making it in one piece, instead of having, as at first, the handle and blade detached.

## Figures and Tables

**Cut No. 1. f1:**
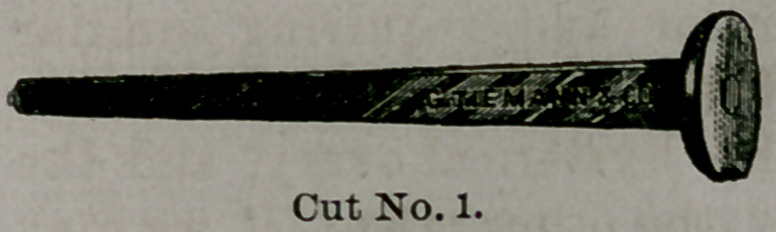


**Cut No. 2. f2:**
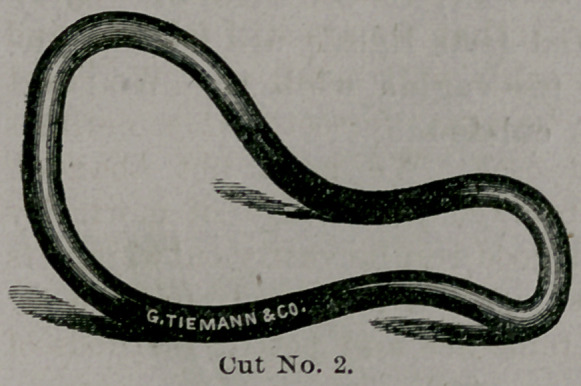


**Cut No. 3. f3:**
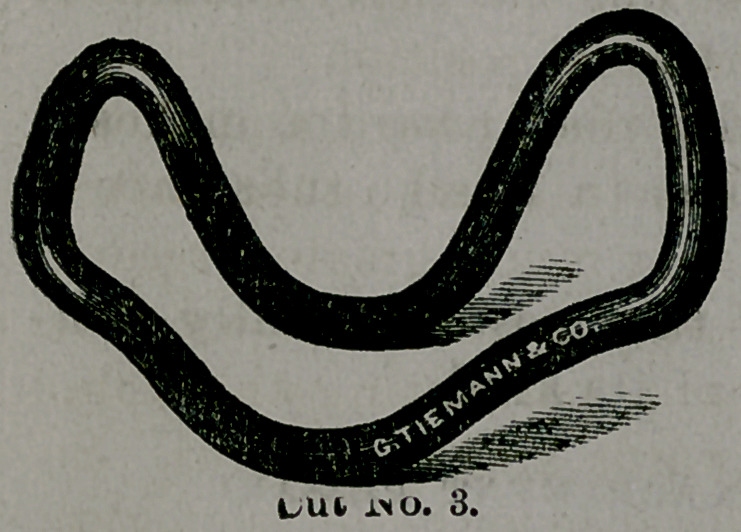


**Cut No. 4. f4:**
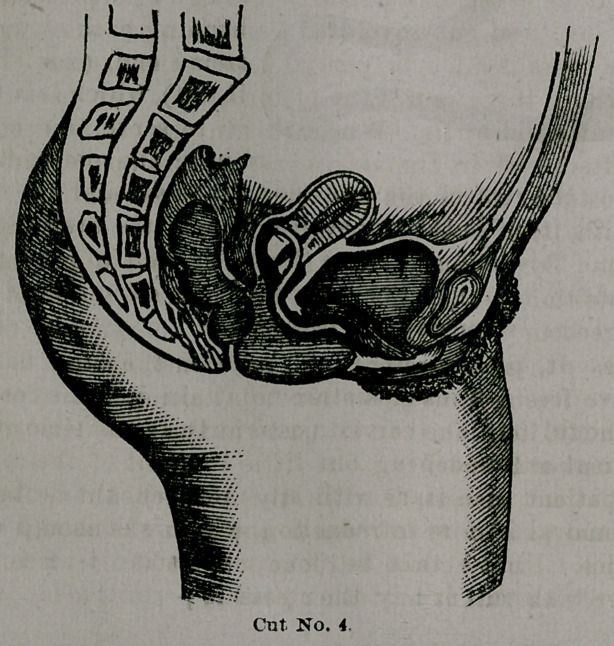


**Cut No. 5. f5:**
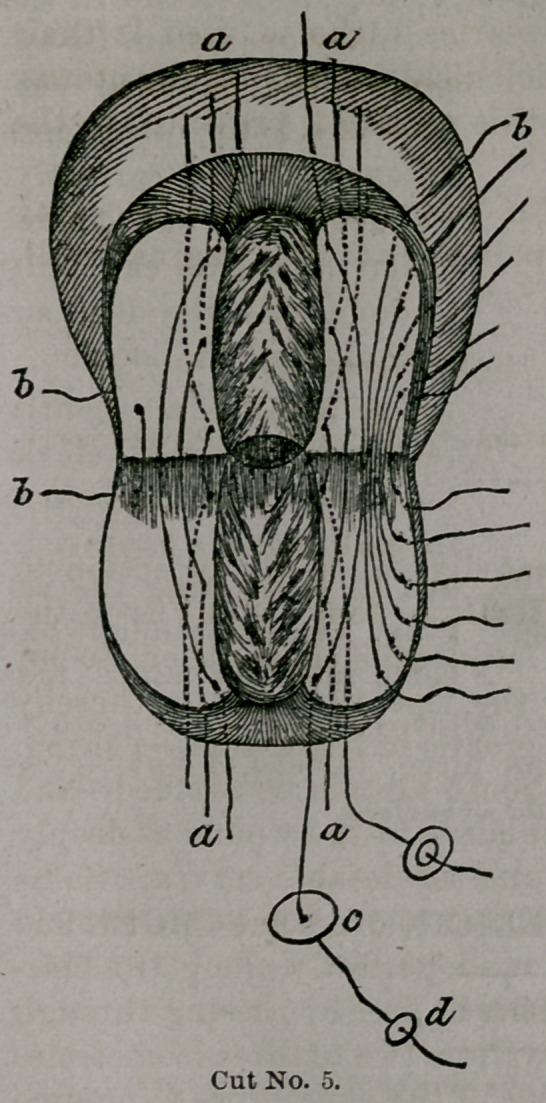


**Cut No. 6. f6:**
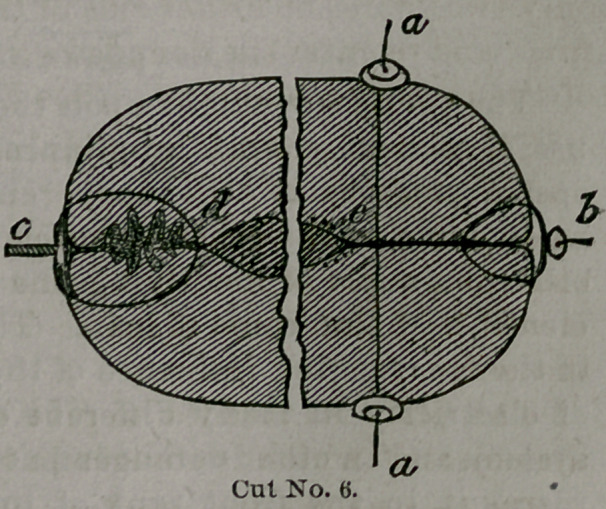


**Cut No. 7. f7:**